# Biochemistry and genetics of ACC deaminase: a weapon to “stress ethylene” produced in plants

**DOI:** 10.3389/fmicb.2015.00937

**Published:** 2015-09-09

**Authors:** Rajnish P. Singh, Ganesh M. Shelke, Anil Kumar, Prabhat N. Jha

**Affiliations:** ^1^Department of Biological Sciences, Birla Institute of Technology and Science (BITS) PilaniPilani, India; ^2^Department of Chemistry, Birla Institute of Technology and Science (BITS) PilaniPilani, India

**Keywords:** ACC, *AcdS*, ethylene, abiotic stress, PGPR

## Abstract

1-aminocyclopropane-1-carboxylate deaminase (ACCD), a pyridoxal phosphate-dependent enzyme, is widespread in diverse bacterial and fungal species. Owing to ACCD activity, certain plant associated bacteria help plant to grow under biotic and abiotic stresses by decreasing the level of “stress ethylene” which is inhibitory to plant growth. ACCD breaks down ACC, an immediate precursor of ethylene, to ammonia and α-ketobutyrate, which can be further metabolized by bacteria for their growth. ACC deaminase is an inducible enzyme whose synthesis is induced in the presence of its substrate ACC. This enzyme encoded by gene *AcdS* is under tight regulation and regulated differentially under different environmental conditions. Regulatory elements of gene *AcdS* are comprised of the regulatory gene encoding LRP protein and other regulatory elements which are activated differentially under aerobic and anaerobic conditions. The role of some additional regulatory genes such as *AcdB* or LysR may also be required for expression of *AcdS*. Phylogenetic analysis of *AcdS* has revealed that distribution of this gene among different bacteria might have resulted from vertical gene transfer with occasional horizontal gene transfer (HGT). Application of bacterial *AcdS* gene has been extended by developing transgenic plants with ACCD gene which showed increased tolerance to biotic and abiotic stresses in plants. Moreover, distribution of ACCD gene or its homolog's in a wide range of species belonging to all three domains indicate an alternative role of ACCD in the physiology of an organism. Therefore, this review is an attempt to explore current knowledge of bacterial ACC deaminase mediated physiological effects in plants, mode of enzyme action, genetics, distribution among different species, ecological role of ACCD and, future research avenues to develop transgenic plants expressing foreign *AcdS* gene to cope with biotic and abiotic stressors. Systemic identification of regulatory circuits would be highly valuable to express the gene under diverse environmental conditions.

## Introduction

Plant growth and productivity are limited by several physiological and environmental factors that include availability of macro and micronutrients, physical and chemical properties of soil, plant genotype and growth conditions. Apart from these factors, plant growth and yield are detrimentally affected by diverse biotic and abiotic factors. The latter include stressors such as salt, low and high temperature, drought, water logging, mechanical wounding, the presence of heavy metals and other organic and inorganic toxic compounds (Gamalero and Glick, 2012). The loss incurred due to these factors is estimated to be >50% for most major crop plants (Boyer, 1982; Bray et al., 2000). Thus, abiotic stresses are the major factors that adversely affect the agricultural productivity worldwide. Therefore, world food production needs to be doubled to cope with the ever-growing demand of the population (Tilman et al., 2002). Problems of biotic and nutritional factors can be overcome using pesticides and biofertilizers, respectively but getting rid of calamities arose due to abiotic factors by non-biological means is highly challenging.

Plants respond to above-mentioned stressors by modulating the level of various hormones which in turn induce expression of stress-related proteins required for protection from the deleterious effects of stressors. One of the most common plant hormone that mediates response to the stressors is ethylene. However, when ethylene is produced more than its threshold level, it turns out as “stress ethylene” which is unfavorable in terms of root/shoot proliferation and other growth parameters and, thus hinders plant growth and development. Effect of stress ethylene in plants can be reduced by certain plant-associated bacteria that possess an enzyme 1-aminocyclopropane-1-carboxylate deaminase (ACCD) (Glick, 2007). ACCD breaks down ACC, an immediate precursor of ethylene, to α-ketobutyrate and ammonia resulting into decrease in level of ethylene in plants which in turn resumes root/shoot growth (Honma and Shimomura, 1978; Glick, 2014). This property of attributing tolerance to abiotic stressors by ACCD activity and some additional mechanisms of plant growth promoting bacteria (PGPB) to ameliorate stresses in host plants are referred as “induced systemic tolerance” (Yang et al., 2009). Thus, PGPB equipped with ACCD activity are of utmost importance in reducing the deleterious effect of environmental stressors (Table 1). It has been established that generation of stress ethylene is central to the effect of various stressors in plants. Therefore, the present review addresses importance of ethylene in plant physiology, details of biochemistry and genetics of ACC deaminase which reduces the level of stress ethylene in plants and relieve from the deleterious effect of environmental stressors.

**Table 1:** **PGPR containing ACC deaminase mediated protection in response to various types of stresses**.

**Bacteria**	**Host plant**	**Stress protection**	**References**
*Achromobacter piechaudii* ARV8	*Lycopersicon esculentum*	Salt	Mayak et al., 2004
*Alcaligenes sp., Bacillus pumilus*	*Brassica napus*	Heavy metals	Belimov et al., 2001
*Burkholderia* sp. J62	*Lycopersicon esculentum*	Lead	Jiang et al., 2008
*B. phytofirmans* PsJN	*Vitis vinifera*	Low temperature	Ait Bakra et al., 2006
*Enterobacter aerogenes* NBRIK24	*Brassica juncea*	Fly-ash soil	Kumar et al., 2008
*E. sakazakii* 8MR5	*Zea mays*	Striga-infested soils	Babalola et al., 2003
*E. cloacae* CAL2	*Brassica napus*	Arsenate	Nie et al., 2002
*Kluyvera ascorbata SUD165*	*Brassica napus*	Nickel	Burd et al., 1998
*K. ascorbata* SUD165/26	*Lycopersicon esculentum*	Lead	Burd et al., 2000
*Pseudomonas putida* UW4	*Lycopersicon esculentum*	Flooding	Grichko and Glick, 2001
*P. fluorescens* YsS6	*Lycopersicon esculentum*	Salt	Ali et al., 2014
*Pseudomonas putida*	*Vigna radiata*	Salt	Mayak et al., 1999
*P. putida* UW4	*Lycopersicon esculentum*	Salt	Yan et al., 2014
*Pseudomonas fluorescens*	*Arachis hypogea*	Salt	Saravanakumar and Samiyappan, 2007
*P. putida*	*Lycopersicon esculentum*	Low temperature	Cheng et al., 2007
*P. fluorenscens* CHA0	*Solanum tuberosum*	Plant pathogen	Wang et al., 2000
*P. fluorenscens* CHA96	*Cucumis sativus*	Plant pathogen	Wang et al., 2000
*P. putida* UW4	*Pinus sabiniana*	Plant pathogen	Nascimento et al., 2013
*P. putida* UW4*, P. putida* HS-2	*Brassica napus*	Nickel	Farwell et al., 2007
*P. fluorescens* ACC-5	*Pisum sativum*	Drough	Zahir et al., 2008
*Pseudomonas sp.*	*Pisum sativum*	Drough	Arshad et al., 2008
*Sinorhizobium sp.* Pb002	*Brassica juncea*	Lead	Di Gregorio et al., 2006
*Variovorax paradoxus*	*Brassica juncea*	Cadmium	Belimov et al., 2005

## Ethylene biosynthesis and role in plant physiology

The ethylene production in plants depends on the environmental condition as well as the severity of various stresses. The recognition of ethylene as a plant growth regulator originated from observation of premature shedding of leaves, geotropism of etiolated pea seedling when exposed to illuminating gas, and ripening of oranges when exposed to gas from kerosene combustion (Pierik et al., 2006; Glick, 2007). Further studies with the analytical techniques like gas chromatography (GC) elaborated its role in plant growth and development. Ethylene at optimal concentration (10 g L^−1^) is essential in functions related to normal growth and development in plants such as formation of adventitious root and root hairs, acceleration of seed germination, breaking seed dormancy etc. (Arshad and Frankenberger, 1990; Jackson, 1991). However, at a higher concentration (25 g L^−1^), it induces defoliation, inhibition of root elongation, inhibition of nodulation in legumes, leaf senescence, leaf abscission, chlorophyll destruction, and epinasty. Therefore, it is imperative to regulate the ethylene production in roots for normal growth and development of the plants.

The ethylene biosynthesis begins with enzyme ACC synthase that converts S-adenosylmethionine (SAM) to 1-aminocyclopropane-1-carboxylic acid (ACC) and 5′-methylthioadenosine (MTA) latter of which is recycled to L-methionine. The next step is the conversion of ACC to ethylene by ACC oxidase. Several isoforms of ACC oxidase has been reported that show differential activity under different physiological conditions (Abeles et al., 1992). Synthesis of ethylene is affected by a number of different factors including temperature, light, nutrition, gravity, and the presence of various types of biological stressors to which the plant may be subjected (Glick, 2007). Regarding a plant's response to stress, an increased level of ethylene is formed in response to the presence of metals, organic and inorganic chemicals, extreme temperature, ultraviolet light, insect attack, phytopathogens (bacteria and fungi), and mechanical wounding. A model proposed for the synthesis of stress ethylene suggests that two peaks of ethylene are observed after stress exposure to plants. The first small peak of ethylene (Robison et al., 2001; Van Loon et al., 2006; Desbrosses et al., 2009) is believed to be responsible for transcription of genes that encode the plant defensive/protective proteins. The second much larger ethylene peak, termed as “stress ethylene” emerged in response to stresses is detrimental to plant growth and initiates processes like senescence, chlorosis and leaf abscission (Glick, 2007). Reduction in the level of stress ethylene by any chemical or biological treatment can significantly lower the magnitude of stress ethylene and decrease stress-induced damage to the plants (Van Loon et al., 2006). As mentioned above, one of the most common and effective mechanisms to reduce the level of stress ethylene is ACCD mediated degradation of ACC. Following sections deal with functioning and molecular aspects of ACC deaminase.

## ACC deaminase: biochemical properties and mode of action

ACC deaminase was first discovered in soil microorganism and shown to convert ACC to ammonia and α-ketobutyrate, both of which further metabolized by a microorganism (Honma and Shimomura, 1978). It is a pyridoxal phosphate-dependent enzyme and approximately 3 mol of enzyme-bound pyridoxal phosphate per mol of an enzyme or 1 mol per trimeric subunit (Honma, 1985) are required for enzyme activity. ACC deaminase was first purified from *Pseudomonas sp. strain* ACP and partially purified from *Pseudomonas chloroaphis* 6G5 (Klee et al., 1991) and *Pseudomonas putida* GR12-2 (Jacobson et al., 1994). Enzyme purified from all three sources appears to have similar molecular mass and form. The native size of 110-112 KDa has been reported from *Pseudomonas sp. strain* ACP and 105 KDa for the enzyme from *P. putida* GR12-2. The enzyme is trimeric in form and has an approximate subunit mass of 36,500 daltons.

The absorption spectra of purified ACC deaminase from *Pseudomonas sp*. show different absorption maxima at 416 and 326 nm at pH 6 and pH 9, respectively (Honma, 1985). It is possible that the 326 nm band seen at pH 9 is the active form of ACC deaminase to which substrates and inhibitors bind (Honma, 1985; Jacobson et al., 1994). The observed K_m_ value of enzyme extracts of different microorganisms at pH 8.5 fall in the range of 1.5–17.4 mM, indicating the low affinity of the enzyme for ACC. The overall efficiency (k_cat_/k_m_) of ACC deaminase is approximately 690 M^−1^S^−1^ following the second order kinetics. The K_m_ value of ACC deaminase for ACC has been estimated for enzyme extracts of microorganism at pH 8.5 (Klee et al., 1991). Enzyme activity of ACCD was evaluated over a wide range of pH in many bacterial species, and the highest activity was observed at pH 8.0–8.5 (Zhao et al., 2003). The optimal temperature for ACC deaminase activity of *P. putida* GR12-2 is 30°C (Glick et al., 1998).

ACC deaminase is an inducible enzyme whose synthesis is induced in the presence of its substrate ACC. The minimum level of the substrate for induction was measured as 100 nM in *Pseudomonas* sp. strain ACP and *P. putida* GR12-2. The induction of ACCD is a complex and slow process. It exhibits activity within the first few hours of induction with the substrate, but the activity decreases gradually after initial induction (Walsh et al., 1981; Jacobson et al., 1994). The basal level of enzyme activity is observed in minimal medium supplemented with ammonium sulfate as a nitrogen source. Honma (1983) demonstrated the induced activity after switching the bacteria from nutrient rich medium to minimal medium supplemented with ACC as sole nitrogen source. It illustrates that the induction of enzyme activity is directly correlated with substrate ACC. Apart from ACC, other amino acids such as L-alanine, DL-alanine, D-serine also induce enzyme activity and induce expression of ACCD to some extent. Moreover, the induced level of enzyme activity by both ACC and aminoisobutyric acid was observed to be same in *Pseudomonas* sp. strain ACP (Honma, 1983). Glick et al. (1998) proposed a model for functioning of bacterial ACC deaminase which states that a significant portion of ACC is exuded from plant roots or seeds, taken up by the soil microbes and hydrolyzed to ammonia and α-ketobutyrate. The uptake and hydrolysis of ACC decrease the amount of ACC outside the plant roots. Furthermore, the equilibrium between the internal and external ACC level is maintained through exudation of more ACC into the rhizosphere. Thus, decrease in the level of ACC affects biosynthesis of the stress hormone ethylene in host plants and stimulate plant growth (Honma et al., 1993; Glick et al., 1998).

Opening of cyclopropane ring of ACC is the main feature of the reaction catalyzed by ACC deaminase. Although the reaction mechanism is not fully understood, nucleophilic addition, and elimination appears to be the most likely routes by which cyclopropane bond is cleaved (Walsh et al., 1981; Ortíz-Castro et al., 2009). ACC deaminase is competitively inhibited by L-isomers of the amino acids such as L-alanine, L-serine, L-homoserine, and L-α aminobutyric acid where the strongest inhibition is seen with L-alanine and L-serine. ACC related compounds such as 2-alkyl -ACC and vinyl-ACC can also function as substrates for ACC deaminase, purified from *Pseudomonas* sp. Strain ACP but the enzyme shows an unusual specificity for D-amino acids and is inactive with any of the L-amino acids or their derivatives (Walsh et al., 1981; Honma, 1985). NMR studies showed that a proton is eliminated from the α-carbon of D-alanine but not from its L-isomer. These findings explain the deamination of D-amino acids and of several β- substituted D-alanines by ACC deaminase and are consistent with the stero-specific cleavage of the cyclopropane ring during ACC deamination (Honma et al., 1993). In the presence of D-alanine, ACC deaminase is inactivated more effectively by the iodoacetamide derivative 1, 5 N-iodoacetamidoethyl-1-aminonapthalene-5-sulfonic acid (1,5-I- AEDANS) than by iodoacetamide. During inactivation, two residues are modified, a thiol group in cysteine residue 162 and the aldimine bond of pyridoxal phosphate with lysine residue 51 (Honma et al., 1993).

## Insight into ACC deaminase catalyzed reaction

Based on three-dimensional structures, pyridoxal phosphate enzymes have been classified into four major types: (a) Tryptophan synthase, (b) aspartate aminotransferase, (c) D-amino acid aminotransferase, and (d) alanine racemase. ACC deaminase is a member of pyridoxal 5-phosphate (PLP) dependent enzymes fitting into the tryptophan synthase family. PLP enzymes catalyze a wide range of metabolic reactions such as transamination, deamination, decarboxylation, and eliminations of β and γ substituent groups. The reaction catalyzed by ACC deaminase differs from other PLP-dependent enzymes as the ring cleavage cannot proceed through α-carbanionic intermediate due to lack of abstractable α-hydrogen atom from the substrate ACC. Two types of reactions can be catalyzed by ACC deaminase for a breakdown of ACC. First, abstraction of hydrogen atom and opening of cyclopropane ring by Lys^51^ mediated series of hydrolytic reactions and second, opening of cyclopropane ring by nucleophilic attack on β-carbon atom of ACC followed by β-proton abstraction at the pro-R carbon by a basic residue Lys^51^ (Zhao et al., 2003).

**Figure 1:**
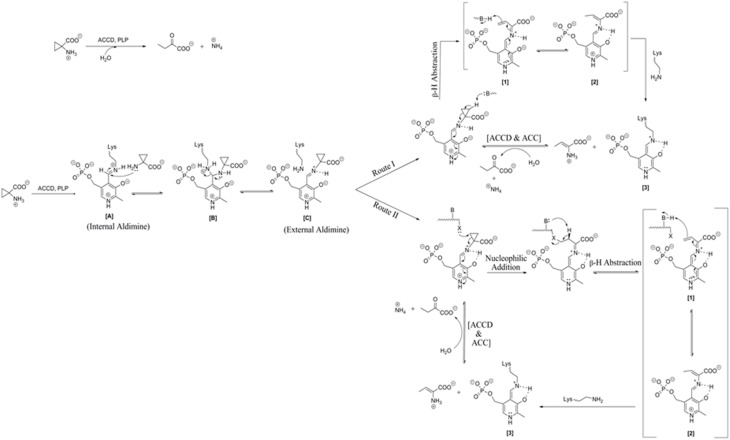
**Reaction mechanism catalyzed by microbial ACC deaminase**. Route I: direct β-H (Hydrogen) abstraction, Route II: Addition of nucleophiles followed by β-H (Hydrogen) abstraction. Modified figure adapted from the source ref. Hontzeas et al. (2006).

The mechanistic action of ACCD on its substrate is depicted in Figure 1. The substrate ACC reacts with internal aldimine [A] resulted from the reaction of PLP (Pyridoxal 5-phosphate) cofactor with Lys residue of ACC deaminase enzyme. This leads to conversion of internal aldimine to external aldimine [C] *via* aminyl intermediate [B] known as trans-aldimination process. These initial mechanistic routes are shared between both proposed mechanisms, i.e., (a) Direct β-hydrogen abstraction and (b) Nucleophilic addition followed by β-hydrogen abstraction.

Ose et al. (2003) proposed that Lys^51^ residue of ACCD causes an initial direct β-hydrogen abstraction of the methylene proton leading to the formation of a quinonoid [1] (Figure 1, Route I). The quinonoid [1] undergoes further electronic rearrangement and protonation to form another quinonoid [2]. This is followed by nucleophilic attack by a basic residue on the protein backbone, which ultimately produces 2-aminobut-2-enoate and a quinonoid [3]. These products reversibly undergo hydrolysis to form 2-oxobutanoate and ammonium, regenerating the internal aldimine. In route II, steps are identical to those proposed for the route I up to the production of external aldimine [C] (Figure 1, Route II). Ring opening is initiated by nucleophilic attack of a basic residue of protein on the pro-S β-carbon of ACC and a nearby second basic residue located on the protein. It follows the removal of a proton from the pro-R β carbon of ACC which results in the formation of a quinonoid. The remaining mechanistic steps are identical to those of the first mechanism (Figure 1, Route II).

## Prevalence of ACC deaminase

The presence of ACC deaminase has been reported in all three domains, i.e., eukarya, bacteria, and archaea. However, ACC deaminase activity is known to be present majorly in different species of bacteria and in some fungi (Table 2). ACCD activity has been found in a wide range of gram positive and gram negative bacteria (Nascimento et al., 2014). It has also been reported in strains of *Pyrococcus horikoshii* (Fujino et al., 2004), a hyperthermophilic archaeon. Among eukaryotes, production of ACCD is well evident in some fungi, which include a few species of yeast such as *Hansenula saturnus* (Minami et al., 1998), *Issatchenkia occidentalis* (Palmer et al., 2007), other fungal species namely *Penicillium citrinum* and *Trichoderma asperellum*, and a stramnopile, *Phytophthora sojae* (Jia et al., 1999; Viterbo et al., 2010; Singh and Kashyap, 2012). Recently, ACCD activity has also been observed in certain plants such as *Arabidopsis thaliana*, poplar, and tomato plant (McDonnell et al., 2009; Plett et al., 2009). Presence of ACCD has been confirmed at the molecular level by amplification and sequence analysis of *AcdS*, a structural gene encoding ACCD. The *AcdS* gene is commonly found in Actinobacteria, Deinococcus-Thermus, three classes of Proteobacteria (α, β, and γ), various fungi belonging to Ascomycota and Basidiomycota, and in some Stramenopiles. Although, the presence of ACCD activity has been demonstrated in bacteria belonging to phyla Chlorobi, Bacteroidetes, and Firmicutes but the genes corresponding to ACCD have not been reported yet (Nascimento et al., 2014). On the contrary, putative *AcdS* genes have been reported in *Meiothermus* and *Phytophthora* based on the sequence similarity but there is no record of ACC deaminase activity in these thermophilic strains.

**Table 2:** **List of microorganism with ACC deaminase activity**.

**Strain**	**ACCD activity (nmol α-KB mg^−1^h^−1^)**	**References**
*Achromobacter xylosoxidans* A551	400	Belimov et al., 2005
*Acidovorax facilis*	0.0007	Belimov et al., 2005
*A. facilis* 4p-6	3080	Belimov et al., 2001, 2005
*A. xylosoxidans* AF302097	151	Belimov et al., 2001
*A. xylosoxidans* Bm1	90	Belimov et al., 2005
*Alcaligenes* sp. AF288728	1172	Belimov et al., 2001
*A. xylosoxidans* AF302096	555	Belimov et al., 2001
*A. xylosoxidans* AF288734	305	Dell'Amico et al., 2008
*Bacillus pumilus* AF288735	760	Belimov et al., 2001
*Burkholderia caryophylli*	598	Shaharoona et al., 2007b
*Enterobacter cloaceae*	295	Nadeem et al., 2010
*E. aerogenes*	341	Nadeem et al., 2007
*Escherichia coli* DH5α/p4U2	285	Shah et al., 1998
*Flavobacterium ferrugineum*	405	Nadeem et al., 2007
*Methylobacterium* sp.	94	Madhaiyan et al., 2006
*Mycobacterium* sp.	1.14	Dell'Amico et al., 2008
*Pseudomonas aeruginosa*	153	Zahir et al., 2009
*P. bathycetes*	501	Nadeem et al., 2007
*P. brassicacearum* AY007428	972	Belimov et al., 2001
*P. chlororaphis*	456	Nadeem et al., 2007
*P. fluorescens*	421	Nadeem et al., 2007
*P. fluorescens* ATCC 17400/pkK-ACC	157	Shah et al., 1998
*P. fluorescens TDK1*	349	Zahir et al., 2009
*P. fluorescens* biotype G	490	Shaharoona et al., 2006a
*P. fluorescens* biotype F	342	Zahir et al., 2008
*P. oryzihabitans* AF288732	890	Belimov et al., 2001
*P. syringae*	440	Nadeem et al., 2007
*P. tolaasii*	1.16	Dell'Amico et al., 2008
*Rhizobium hedysari* ATCC 43676	20	Ma et al., 2003b
*R. leguminosarum*128C53K	5	Belimov et al., 2001, 2005
*R. leguminosarum* 99A1	430	Ma et al., 2003a
*Rhodococcus* sp. AF 288731	833	Belimov et al., 2001
*Rhodococcus* sp. strain Fp2	7320	Belimov et al., 2001, 2005
*Rhodococcus* sp. strain 4N-4	12,970	Belimov et al., 2001, 2005
*Serratia ficaria*	326	Nadeem et al., 2010
*S. proteamaculans*	276	Zahir et al., 2009
*S. quinivirans* SUD165	12	Belimov et al., 2001, 2005
*Variovorax paradoxus* 3P-3	3700	Belimov et al., 2001, 2005
*V. paradoxus* 5C-2	4322	Belimov et al., 2001, 2005
*V. paradoxus* 2C-1	3588	Belimov et al., 2001, 2005
*V. paradoxus* sp.	1805	Belimov et al., 2005

We analyzed the prevalence of *AcdS* gene in IMG (Integrated microbial genomes) database (http://img.jgi.doe.gov/) from Joint genome Institute (JGI) using locus tag search corresponding to *AcdS* of *P. putida* UW4. The *AcdS* sequences having more than 1000 bp were chosen for further analysis. Altogether, 485 strains belonging to different genera including *Acidovorax, Bordetella, Brenneria, Burkholderia, Collimonas, Cupriavidus, Curvibacter, Dickeya, Herbaspirillum, Halomonas, Lonsdalea, Methylibium, Pantoea, Phytophthora, Polaromonas, Pseudomonas, Ralstonia, Serratia, Tatumella, Variovorax*, and *Xenophilus*, showed presence of *AcdS* gene. Important species belonging to these genera are listed in Supplementary Table 1. These data were extracted from the genomic sequences of organisms from different ecological niches including a human host, bulk soil, plants, and water. For the presence of gene encoding ACCD in other domains (eukarya and archaea), gene search was conducted using the product name as a criterion for the search. It revealed that very few members of archea showed the presence of ACCD gene. It includes strains of *Archaeoglobus fulgidus, Pyrococcus abyssi, Pyrococcus furiosus*, and *Thermococcus nautili.* Analysis of metagenomic database revealed the presence of genes encoding ACCD in various kingdoms, i.e., animalia, chromalveolate, fungi, and plantae of domain Eukarya (Table 3). It suggested that among domain Eukarya, ACCD gene is prevalent in members of phylum Ascomycota and Basidiomycota. The IMG database extends our knowledge of the existence of ACCD gene in other higher plants from kingdom plantae which include soybean, potato, maize, and castor oil plants. The presence of ACCD encoding genes in several members of kingdom Animalia seems to be intriguing as no obvious role of ACCD in animals is known (Table 3). The presence of ACCD gene in some pathogenic bacteria associated with human and other animals indicates that ACCD may be required for some other unknown function or these bacteria may earlier be plant-associated which later on evolved to colonize animals and other kingdoms as well. Moreover, both bacterial and fungal ACC deaminase shares a common origin and belongs to pyridoxal phosphate-dependent enzyme related to tryptophan-synthase family.

**Table 3:** **Distribution of ACC deaminase in domain Eukarya**.

**Kingdom**	**Phylum**	**Species**
Animalia	Porifera	*Amphimedon queenslandica*
	Chordata	*Branchiostoma floridae, Ciona intestinalis, Oikopleura dioica*
	Annelida	*Capitella teleta*
	Cnidaria	*Nematostella vectensis*
	Placozoa	*Trichoplax adhaerens Grell-BS-1999*
Chromealveolata	Haptophyta	*Emiliania huxleyi*
	Stramnopile	*Saprolegnia parasitica, Phytophthora ramorum*
	Apicomplexa	*Theileria annulata*
Fungi	Ascomycota	Arthroderma benhamiae^*^, Aspergillus clavatus^*^, A. flavus^*^, A. fumigatus, Beauveria bassiana, Cercospora sojina, Coccidioides posadasii^*^, Fusarium oxysporum, Neosartorya fischeri^*^, Penicillium marneffei^*^, Talaromyces stipitatus^*^, Trichophyton verrucosum^*^
	Basidiomycota	*Cryptococcus neoformans, Gymnopus luxurians, Schizophyllum commune*
Plantae	Lycopodiophyta	*Selaginella moellendorfii*
	Magnoliophyta	Ricinus communis^*^, Zea mays, Glycine max, Solanum tuberosum
	Chlorophyta	*Volvox carteri*

* Putative proteins have been found.

## Genetics and expression of ACC deaminase

### ACC deaminase gene

Gene *AcdS* encoding ACC deaminase have been detected in several bacterial and fungal genera as discussed above. More recently, ACC deaminase has been found in wide range of gram-negative bacteria (Belimov et al., 2001; Wand et al., 2001; Hontzeas et al., 2004; Tak et al., 2013), Gram-positive bacteria (Belimov et al., 2001; Timmusk et al., 2011), rhizobia (Ma et al., 2003b; Uchiumi et al., 2004), endophytes (Sessitsch et al., 2002; Rashid et al., 2012), and fungi (Jia et al., 1999). Putative ACC deaminase gene have also been reported several species including *R. leguminosarum bv. Trifoli* (Itoh et al., 1996) and *Mesorhizobium loti* MAFF303099 (Kaneko et al., 2002). However, the expression level of ACC deaminase varies from one organism to another. Using a universal pair of primers, a segment of *AcdS* gene has been amplified and analyzed in several environmental isolates (Hontzeas et al., 2005). Several pair of primers has been designed by various researchers to detect the presence of *AcdS* gene in bacteria (Duan et al., 2009; Jha et al., 2012). Complete genetic makeup and function of ACCD gene has been well characterized in only a few bacterial species (Duan et al., 2013). We observed from the data recovered from IMG that nucleotide sequences of *AcdS* gene is very close to other genes namely dcyD and yedO which encode for another PLP-dependent enzyme D-cysteine sulfhydralase. This observation is supported by previous reports where some genes previously identified to encode ACC deaminase were found to encode D-cysteine desulfhydrase activity (Riemenschneider et al., 2005). To differentiate sequences of D-cysteine desulfhydrase from ACC deaminase, Nascimento et al. (2014) analyzed *AcdS* sequences for key protein residues namely Lys51, Ser78, Tyr295, Glu296, and Leu322, known to be important for ACC deaminase activity using *Pseudomonas* sp. UW4 as a reference. Any change in residues at given locations were considered likely to represent D-cysteine desulfhydrase.

Except few, *AcdS* gene in the majority of bacterial species is chromosomal DNA-borne. In symbiotic bacteria *M. loti* (symbiont of *lotus* spp.), ACC deaminase gene is associated with the nitrogen fixation genes and might be regulated by NifA which is known to activate *nif* gene expression in association with the product of *rpoN* gene (Ma et al., 2003a). Moreover, only a small fraction of putative *AcdS* gene has been shown to encode active enzyme (Glick et al., 2013).

### Regulation of ACC deaminase

**Figure 2:**
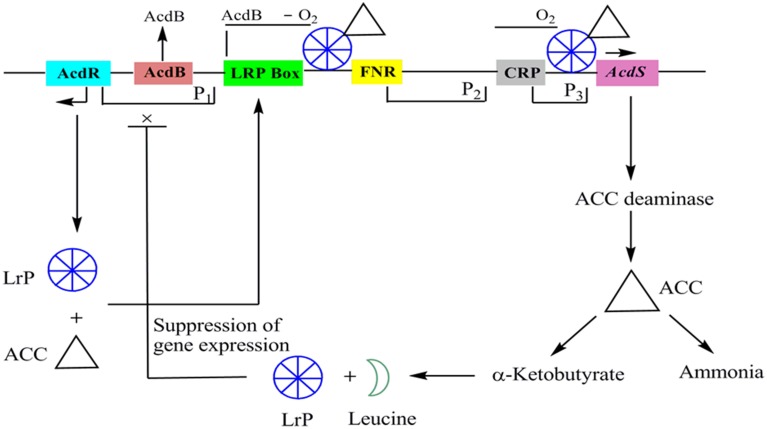
**The regulatory circuits of *AcdS* gene expression in *Pseudomonas putida* UW4 and other related bacteria**. AcdR, regulatory gene for ACC deaminase; AcdB: encoding for glycerophosphoryl diester phosphodiesterase; LRP, Leucine responsive protein; FNR: fumarate nitrate reductase protein; CRP, c-AMP receptor protein; *AcdS*, gene for encoding ACC deaminase.

*AcdS* is highly regulated and expresses differentially depending on presence or absence of oxygen, concentration of substrate, and accumulation of products. Except few, mechanism of regulation of *AcdS* gene in different bacterial genera is not well understood. A model for the regulation of ACC deaminase gene in *P. putida* UW4 (earlier known as *Enterobacter clocae* UW4) has been proposed by Li et al. (2000). Regulatory elements for the expression of ACC deaminase gene consist of regulatory gene *AcdR* located 5′ upstream of ACC deaminase structural gene (*AcdS*), promoter regions for binding of regulatory proteins like Lrp box for binding of Lrp protein, AcdB box for binding regulatory protein *AcdB*, FNR box for binding of fumarate and nitrate reductase protein and, CRP box for binding of cAMP receptor protein. In the presence of ACC, LRP forms an active octamer that binds to a complex of ACC and another protein *AcdB* (Cheng et al., 2008). *AcdB* encodes for the glycerophosphoryl diester phosphodiesterase and form complex with ACC. This triparental complex activates transcription of *AcdS* by binding to its promoter region (Li and Glick, 2001). The role of *AcdB* in *AcdS* expression has not been observed in other bacteria characterized for *AcdS* gene expression. ACC deaminase gene is negatively regulated by leucine which is synthesized from α-ketobutyrate, a breakdown product of ACCD catalyzed reaction. As the concentration of leucine increases, it favors formation of inactive LRP dimer form which leads to switching off the transcription of *AcdS* gene (Figure 2).

Regulatory machinery for *AcdS* expression varies in different species. Results of IMG database analysis showed that presence of *AcdR* encoding LRP or its homologous sequences is present in the majority of bacteria. In *Bradyrhizobium japonicum* USDA 110 and *Rhizobium leguminosarum bv. Viciae* 128 C53K also, LRP like protein and σ70 promoter are involved in regulation of *AcdS* gene (Kaneko et al., 2002; Ma et al., 2003a). The phylogenetic analysis of *AcdR* gene suggested that *AcdS* and *AcdR* were evolved in a similar manner. In *Burkholderia* sp. CCGE 1002 and *Burkholderia phymatum* STM 815, there is no *AcdR* gene but it has two copies of *AcdS* gene instead, one on the megaplasmid and other on the second chromosome. These shreds of evidence suggest the genomic rearrangement events or gene insertion event in smaller replicons. Regulatory regions of ACC deaminase gene from some bacteria such as *Variovorax parrdoxus* 5C2 and *Achromobacter xylosoxidans* A551 does not contain all these regulatory elements described for *P. putida* UW4.

**Figure 3:**
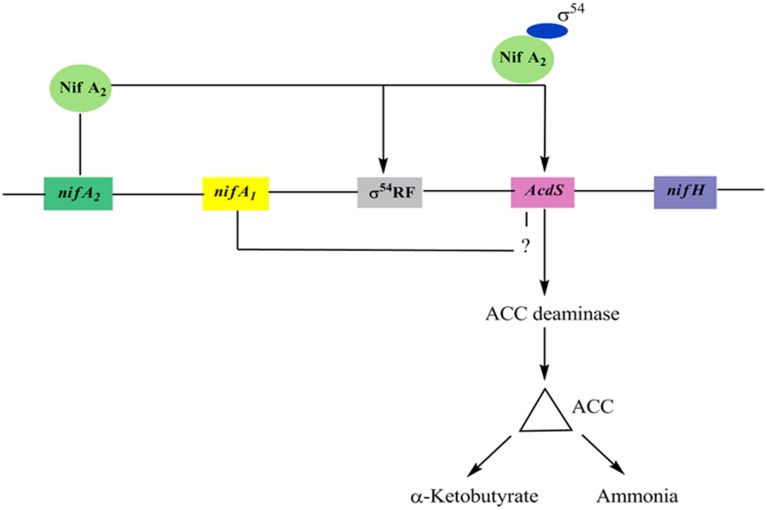
**A model for *AcdS* gene regulation in nitrogen fixing *Mesorhizobium* sp. Expression of *AcdS* is positively regulated by NifA_2_ protein which binds to σ^54^ and switch on transcription of *AcdS* gene**. NifA_1_ is also required in regulation of *AcdS* but its role is not well-understood.

In *M. loti*, the upstream elements of *AcdS* and *nifH* contain *nifA1* and *nifA2* (regulatory N_2_ fixing unit) and σ^54^ RNA polymerase sigma recognition site. It was assumed that expression of ACC deaminase in *M. loti* required the symbiotic nitrogen fixing regulatory gene *nifA2* (Nukui et al., 2006). The *nifA2* encoded protein NifA2 interact with σ^54^ RNA polymerase favoring *AcdS* transcription. The *nifA1* also affect the transcription of *AcdS* gene up to some extent. however, its role in the expression of *AcdS* is not properly understood (Nukui et al., 2006) (Figure 3). The expression of *AcdS* gene in root nodules reduces the harmful effect of ethylene induced senescence and elevates the concentration of fixed nitrogen in nodules. ACC deaminase activity is generally assayed in free-living conditions but in *M. loti*, the activity was detected only in symbiotic nodules (Uchiumi et al., 2004). Uchiumi et al. (2004) have reported that mlr5932, an up-regulated gene in Bacteroides, encodes ACC deaminase, which is involved in enhancing nodulation in *Lotus japonicus* plants. It is to be noted that unlike free-living bacteria, ACC deaminase produced by nodule forming *Rhizobia* does not lower the ethylene level throughout the plant and may not be used to protect plants from various stress. Also, the level of ACCD produced in the nodule is only 2–10% of ACCD produced by free-living bacteria.

In many *Actinobacteria* and *Meiothermus*, GntR protein coding gene is found next to *AcdS* gene. These evidence indicate a possibility that some downstream elements are also involved in regulation of ACC deaminase expression. In some members of these genera, the absence of promoter region strongly suggests that the interaction of *AcdS* gene and some downstream element next to *AcdS* gene is involved in the regulation of *AcdS* gene transcription. In certain species of *Actinobacteria* and *Proteobacteria* like *Brenneria* sp. EniD312, *Burkholderia xenovorans* LB4000 and *Pantoea sp*. At-9B, LysR family of transcription regulatory elements, are found in close vicinity of *AcdS* gene. However, the exact mechanism of regulation of ACC deaminase in these organisms is still poorly known. Additional genetic and biochemical studies are necessary to understand the mechanism of ACC deaminase regulation and functioning in different bacterial genera.

Putative ACC deaminase gene in *M. loti* MAFF303099 does not contain any regulatory elements nor display any enzyme activity when it is induced by ACC (Ma et al., 2003b). Expression of ACC deaminase in *R. leguminosarum bv. viciae* 128C53K is induced by ACC concentrations as low as 1 μM (Ma et al., 2003b). Experimental evidence suggested that introducing ACC deaminase gene as well as its regulatory gene from *R. leguminosarum bv. Viciae* 128C53K to a strain of *Sinorhizobium meliloti* showed greater efficiency in nodulating *Medicago sativa* (alfalfa) (Ma et al., 2004) and latter strain was more competitive in nodulation than the wild type (Ma et al., 2004).

### Evolution of ACC deaminase gene

**Figure 4:**
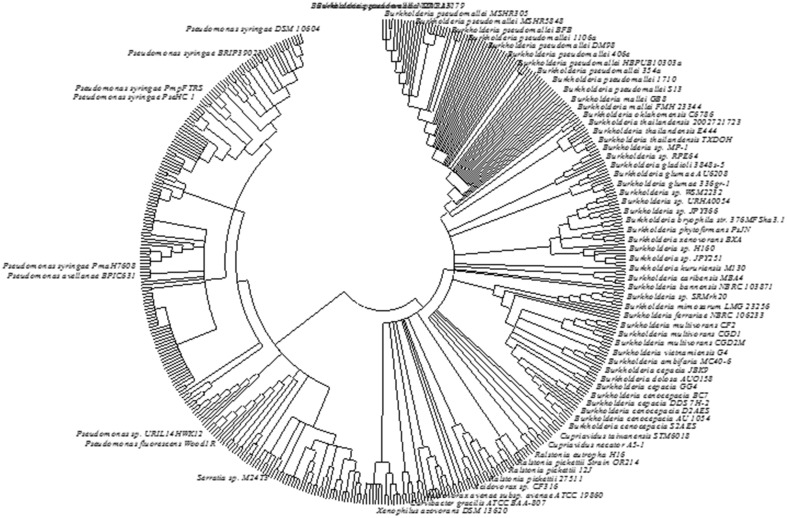
**The phylogentic tree constructed from *AcdS* gene sequence of different bacterial strains**. The sequence data obtained from IMG database of JGI were used. The evolutionary history was inferred using the Neighbor-Joining method. The bootstrap consensus tree inferred from 500 replicates was taken to represent the evolutionary history of the taxa analyzed. Branches corresponding to partitions reproduced in less than 50% bootstrap replicates are collapsed. The evolutionary distances were computed using the Maximum Composite Likelihood method and were in the units of the number of base substitutions per site. The analysis involved 465 nucleotide sequences. All positions containing gaps and missing data were eliminated. There were a total of 958 positions in the final dataset. Label of bacterial strains/species showing values lower than 1% was hidden. Evolutionary analyses were conducted in MEGA6.0.

Phylogenetic analysis based on *AcdS* gene and protein sequence from different microbial species has been conducted to study the evolution of ACC deaminase gene. Available sequence data revealed that the most of the ancient bacteria belonging to Actinobacteria and Deinococcus-Thermus possess *AcdS* gene in their primary and unique chromosome. The *AcdS* gene in many α-proteobacteria is also found in the chromosomal DNA. The presence of *AcdS* gene has also been observed in plasmid observed on a plasmid DNA in a few bacteria which might have resulted from an event of extensive gene transfer from primary chromosome to plasmid between the members of α-proteobacteria. Location of *AcdS* gene in the second chromosome of *Burkholderia* and in plasmids of *Pseudomonas* reflects the intragenomic transfer of *AcdS* genes from primary chromosome to plasmids. On the other hand, the presence of *AcdS* gene sequence in some fungi like *Fomitopsis pinicola* FP-58527 belonging to Basidiomycota suggested having a bacterial origin. The monophyletic origin of ACC deaminase is evident from the conservation of *AcdS* protein sequences from fungi, Actinobacteria, and α-proteobacteria signify. The presence of *AcdS* gene in some fungal classes and in members of *Stramenopiles* suggest that horizontal gene transfer (HGT) resulted in the transfer of *AcdS* not only between bacteria but also between different domains. To gain additional knowledge about the evolution of ACC deaminase gene, multiple *AcdS* gene sequences and protein sequences were aligned. Based on phylogenetic studies, it was assumed that ACC deaminase belongs to a broader group of pyridoxal phosphate-dependent enzymes that share a common ancestor. It might be possible that this enzyme was originated as a consequence of specific mutation in its ancestral enzyme gene (Nascimento et al., 2014). Recently, based on alignment of multiple *AcdS* sequence from diverse species, Nascimento et al. (2014) inferred that the continuous vertical transmission of *AcdS* genes might be responsible for the presence of *AcdS* gene in bacteria that are not associated with ACC producing organism. Our analysis of *AcdS* using IMG database and subsequent construction of phylogenetic tree also confer that evolution of *AcdS* gene resulted from vertical gene transfer with occasional HGT. A consensus bootstrap tree was constructed using 465 *AcdS* sequences obtained from IMG database which showed that accept few, *AcdS* of similar species clustered together supporting the above inference (Figure 4). However, many earlier studies suggested a role of HGT in the evolution of *AcdS* gene. Intragenomic *AcdS* transfer might play a role in HGT transfer as well as the divergence of *AcdS* gene. Furthermore, these intragenomic transfers may also lead to loss of the gene in many organisms. Phylogenetic analysis of several *AcdS* gene from ACC deaminase bacteria such as *Variovorax paradoxus 5C-2, V. paradoxus 3P-3, and Achromobacter spp.* indicated that the gene evolved through HGT (Hontzeas et al., 2005). Moreover, the presence of *AcdS* gene in the symbiotic island of many *mesorhizobium spp*. like *M. loti* R7A, *M. australicum* WSM2073T, *M. opportunistum* WSM2075T confirms the horizontal transfer of *AcdS* gene (Nascimento et al., 2012b). Even though most of the phylogenetic studies of *AcdS* gene has been done on proteobacteria, presence of ACC deaminase enzyme have also been reported in Actinobacteria (Hontzeas et al., 2005), Firmicutes (Ghosh et al., 2003), and Bacteroidetes (Maimaiti et al., 2007).

Prigent-Combaret et al. (2008) reported that like *AcdS, AcdR* also have evolved through HGT. Owing to horizontal (lateral) gene transfer, the juxta-position of *AcdS* and *AcdR* gene has occurred in *Agrobacterium tumefaciens* d3, *B. japonicum* USDA 110, *R. leguminosarum bv. Viciae* 128C53K, *A. xylosoxidans* A551 (Trott et al., 2001; Kaneko et al., 2002; Hontzeas et al., 2005). However, it is not clear that *AcdR* gene was inherited along with the *AcdS* gene or independently. The current knowledge of the phylogeny and evolution of *AcdS* and *AcdR* gene is still incomplete. The presence of *AcdR* in juxta-position to *AcdS* gene in most proteobacteria acquired through a coupled evolution and transmission of these genes. Therefore, more research is required for elucidating the role of protein encoding genes located in the vicinity to *AcdS*, which can focus on a different mechanism of ACC deaminase regulation.

### Ecological significance of ACC deaminase bacteria

The ACC deaminase activity is one of the most common traits among plant growth promoting rhizobacteria (Honma and Shimomura, 1978; Glick, 2005). ACCD bacteria exert its beneficial effect by protecting from the deleterious effect of environmental stressors (Glick, 2014), delay senescence (Ali et al., 2012), exhibit biocontrol activity against variety of phytopathogens in certain plants (Hao et al., 2011), and favor nodulation in legume plants (Nascimento et al., 2012a). Role and importance of bacterial ACCD in plant growth have been described in previous sections. Variation of ACC deaminase activity among microbial species at extreme environmental conditions might be useful in phytoremediation at unusual environmental sites or conditions (Glick, 2005). ACC deaminase bacteria assist associated plants in phytoremediation by biotransformation of toxic elements, rhizodegradation mediated by root exudates, and/or detoxification of heavy metals that allow host plants to survive under adverse conditions. Rhizospheric bacterial community with ACC deaminase can enhance the rate of rhizo-remediation by increasing the root system of the plant as well as increased access to soil by roots. It results in enhanced uptake of inorganic contaminants through modification of root architecture and root uptake system of the plant. Belimov et al. (2005) reported a positive correlation between the increment of bacterial ACC deaminase activity following the accumulation of cadmium in plant tissue and enhanced root growth. Similarly, Rodriguez et al. (2008) observed the enhanced growth of tobacco plants and substantial accumulation of metals from nickel contaminated soil following inoculation of *P. putida* HS-2.

The presence of ACC deaminase in human pathogenic *Burkholderia cenocepacia* J 2315 as well as in plant pathogenic fungi like *Aspergillus spp.* and *Myceliophthora thermophile* suggests that ACC deaminase might play a role in the ecological fitness of these micro-organisms. Role of ACC deaminase in endophytic fungi *P. citrinum* was investigated by Jia et al. (2000) who found accumulation of ACC during mycelial growth and subsequent degradation of ACC by ACC deaminase when the mycelial growth reached the maximum. Importance of ACCD in human pathogenic bacteria is not known, but its role in the pathogenesis of plant pathogens has been studied to some extent. For plant pathogenic microorganisms, it is assumed that production of ACC deaminase may help microbe to overcome ACC mediated plant responses. The presence of ACC deaminase bacteria in the close vicinity of fungal strains might have a role in increased fungal primordial proliferation by reducing ACC levels. Thus, an association of ACC deaminase bacteria plays a significant role in fungal colonization in the extreme soil. Additional advantage of these bacteria is the ability to degrade ACC providing extra nutrients to plant (Nascimento et al., 2014).

### Transgenic plants with ACC deaminase activity

The growth enhancement of plant by ACC deaminase bacteria has motivated scientists to transfer this gene into plants as future approach to minimize the deleterious effect of ethylene in plants subjected to adverse environmental conditions (Grichko and Glick, 2001; Robison et al., 2001; Nie et al., 2002). A transgenic Petunia hybrid with ACC deaminase gene maintains a significantly reduced amount of ACC in pollen cells (Lei et al., 1996). Similarly, the transgenic canola plants (*Brassica napus*) with ACC deaminase perform better growth under salinity stress compared to non-transgenic plant (Sergeeva et al., 2006). In a premier study, Reed et al. (1995) transformed two tomato cultivars with *AcdS* gene of *Pseudomonas chlororaphis* which resulted in lengthened duration of fruit ripening as well as significant reduction of stress ethylene production compared to parental line. Klee and Kishore (1992) also observed a significant reduction of ethylene production (77%) and delayed in senescence in tobacco and tomato plants transformed with bacterial *AcdS.* The tomato plant (*Lycopersicon esculentum*) expressing *AcdS* gene of *Pseudomonas* sp. 6GS exhibited reduced ethylene synthesis up to 90% (Klee et al., 1991). A large number of transgenic plants with foreign *AcdS* gene have been genetically engineered to reduce the deleterious ethylene levels in plants (Grichko and Glick, 2000; Robison et al., 2001; Sergeeva et al., 2006; Farwell et al., 2007; Zhang et al., 2008). However, there is a limited report of the performance of transgenic plant containing *AcdS* gene under field condition (Farwell et al., 2006, 2007). Furthermore, future research should focus on (i) field performance of transgenic plant, (ii) their survival and yield under diverse condition, and (iii) genetic re-arrangement for target gene identification for gene insertion and deletion.

## Conclusion and future prospects

Increasing global warming and environmental pollution in the present scenario have highly affected the agricultural production which is confronted with induced stress generated by both biotic and abiotic factors. Bacteria with ACCD activity are able to mediate the enhanced resistance to biotic stressors and increased tolerance to abiotic stresses in their associated plants. Thus, these bacteria have the potential to promote plant growth under adverse environmental conditions. Therefore, it necessitates exploration of efficient PGPB with strong ACCD activity which can colonize plants effectively and increase plant productivity under actual farming conditions. ACCD is an inducible enzyme which expresses on the availability of ACC and regulated differentially by various physiological factors. This enzyme is tightly regulated. However, precise mechanisms of its regulation are understood only in few bacteria. Lack of information about the regulatory mechanism for expression of ACCD gene in the majority of organisms is a major constraint. The understanding regulatory circuit of ACCD gene will be helpful in the optimal exploitation of ACCD bacteria for enhancing plant productivity. Based on current knowledge, a very few plants like *Arabidopsis*, tomato, poplar have been reported to contain *AcdS* gene. Therefore, future research needs to explore *AcdS* gene in other plant species. The role of ACCD in the plant has been evident in fruit development in tomato and other plants. However, their direct role in stress is not well characterized. Therefore, distribution of ACCD gene in different plants and its possible role in stress amelioration needs to be investigated in detail. Development of transgenic plant overexpressing foreign *AcdS* gene could be used to overcome stress ethylene generated through stress conditions. Moreover, the exact role of ACCD in bacteria also needs to be investigated in greater detail. The presence of ACCD gene in bacteria other than plant-associated bacteria intrigues its role and suggests the possibility for an alternative function.

### Conflict of interest statement

The authors declare that the research was conducted in the absence of any commercial or financial relationships that could be construed as a potential conflict of interest.
