# Corrigendum: Biochemistry and genetics of ACC deaminase: a weapon to “stress ethylene” produced in plants

**DOI:** 10.3389/fmicb.2015.01255

**Published:** 2015-11-06

**Authors:** Rajnish P. Singh, Ganesh M. Shelke, Anil Kumar, Prabhat N. Jha

**Affiliations:** ^1^Department of Biological Sciences, Birla Institute of Technology and Science (BITS) PilaniPilani, India; ^2^Department of Chemistry, Birla Institute of Technology and Science (BITS) PilaniPilani, India

**Keywords:** ACC, Acds, ethylene, abiotic stress, PGPR

We mistakenly did not cite the reference Gontia-Mishra et al. (2014) in the text. Please find the following corrected paragraph with the given citation in the appropriate place. The full citation is also written below for inclusion in the reference list.

As reviewed in Gontia-Mishra et al. (2014), among eukaryotes, production of ACCD is well evident in some fungi, which include a few species of yeast such as *Hansenula saturnus* (Minami et al., 1998), *Issatchenkia occidentalis* (Palmer et al., 2007), other fungal species namely *Penicillium citrinum* and *Trichoderma asperellum*, and a *stramnopile, Phytophthora sojae* (Jia et al., 1999; Viterbo et al., 2010; Singh and Kashyap, 2012). Recently, ACCD activity has also been observed in certain plants such as *Arabidopsis thaliana*, poplar, and tomato plant (McDonnell et al., 2009; Plett et al., 2009).

## Conflict of interest statement

The authors declare that the research was conducted in the absence of any commercial or financial relationships that could be construed as a potential conflict of interest.
