# Supplementary material for: Biochemistry and genetics of ACC deaminase: a weapon to “stress ethylene” produced in plants
Source: Front Microbiol. 2015 Sep 9;6:937. doi: 10.3389/fmicb.2015.00937 (PMC4563596; doi:10.3389/fmicb.2015.00937)
Supplement: Supplementary file 1 [file Table1.DOCX]

**Suppl. Table 1**: **Bacterial species known to possess *AcdS* gene. The data has been extracted from genomic and metagenomic database available on JGI-IMG database. Search was made using locus tag of standard strain UW4 of *Pseudomonas sp.***

| **Species** |
| --- |
| *Acidovorax avenae* |
| *Bordetella sp.* |
| *Brenneria salicis* |
| *Burkholderia ambifaria* |
| *Collimonas fungivorans* |
| *Cupriavidus basilensis* |
| *Curvibacter gracilis* |
| *Dickeya zeae* |
| *Halomonas boliviensis* |
| *Herbaspirillum lusitanum* |
| *Herbaspirillum sp.* |
| *Lonsdalea quercina quercina* |
| *Methylibium petroleiphilum* |
| *Pantoea sp.* |
| *Phytophthora ramorum* |
| *Polaromonas sp.* |
| *Polaromonas sp.* |
| *Pseudomonas avellanae* |
| *Ralstonia eutropha* |
| *Serratia sp.* |
| *Tatumella ptyseos* |
| *Variovorax sp.* |
| *Xenophilus azovorans* |
